# Ibandronate Treatment Before and After Implant Insertion Impairs Osseointegration in Aged Rats with Ovariectomy Induced Osteoporosis

**DOI:** 10.1002/jbm4.10184

**Published:** 2019-03-06

**Authors:** Ethan M. Lotz, David J. Cohen, Regan A. Ellis, Jennifer S. Wayne, Zvi Schwartz, Barbara D. Boyan

**Affiliations:** ^1^ Department of Biomedical Engineering College of Engineering Virginia Commonwealth University Richmond VA 23284 USA; ^2^ Department of Periodontics University of Texas Health Science Center at San Antonio San Antonio TX 78229 USA; ^3^ Wallace H. Coulter Department of Biomedical Engineering Georgia Institute of Technology Atlanta GA 30332 USA

**Keywords:** Osseointegration, Osteoporosis, Bisphosphonate, Rat, Titanium

## Abstract

Excessive decreases in bone volume (BV) and bone mineral density (BMD) can lead to osteoporosis, potentially hindering implant osseointegration. Bisphosphonates are commonly used to combat osteoporosis by slowing osteoclast‐mediated resorption; however, functional osteoclasts are integral to bone remodeling and, thus, implant osseointegration, potentially contraindicating bisphosphonate use during implantation. To optimize the use of implant technologies in patients with compromised bone structure and metabolism, we need a more complete understanding of the biological response to surface design. The goal of this study was to assess the effects of osteoporosis and bisphosphonates on osseointegration of titanium (Ti) implants with microstructured surfaces, which have been shown to support osteoblast differentiation *in vitro* and rapid osseointegration *in vivo*. Forty, 8‐month‐old, virgin, female CD Sprague Dawley rats underwent ovariectomy (OVX) or sham (SHOVX) surgery. After 5 weeks, animals were injected subcutaneously with either the bisphosphonate (BIS), Ibandronate (25 µg/kg), or phosphate‐buffered saline (PBS) every 25 days. 1 week after the initial injection, Ø2.5mm × 3.5mm microrough (SLA; grit‐blasted/acid etched) implants were placed transcortically in the distal metaphysis of each femur resulting in four groups: 1) SHOVX+PBS; 2) SHOVX+BIS; 3) OVX+PBS; and 4) OVX+BIS. After 28d, qualitative properties of the bone and implant osseointegration were assessed using micro‐computed tomography (microCT), calcified histomorphometry (Van Gieson's stain), and removal torque testing. microCT revealed decreased bone volume in OVX rats, which was slowed by bisphosphonate treatment. Reduced bone‐to‐implant contact (BIC) was evident in OVX+PBS compared to SHOVX+PBS. Although BV/TV was increased in OVX+BIS compared to OVX+PBS, bisphosphonate treatment had no effect on BIC. Removal torque testing revealed a higher maximum torque, torsional stiffness, and torsional energy in SHOVX compared to OVX with no effects due to bisphosphonate treatment. Our results show that osseointegration is decreased in osteoporotic animals. Ibandronate halts the progression of osteoporosis but does not enhance osseointegration. © 2019 The Authors. *JBMR Plus* Published by Wiley Periodicals, Inc. on behalf of American Society for Bone and Mineral Research

## 1. Introduction

Sufficient bone volume (BV) and bone mineral density (BMD) are two of the most important patient factors for predicting the long‐term success of dental and orthopaedic implant osseointegration, which is defined as the direct anchorage of an implant to mature bone tissue without the growth of fibrous tissue.^1^, ^2^, ^3^ These factors significantly diminish with age and their reduction is exacerbated by certain factors including postmenopausal estrogen deficiency. Osteoporosis is also characterized by excessive decreases in BMD and BV as a result of increased rates of bone turnover. An estimated 53.6 million U.S. adults over the age of 50 were affected by osteoporosis or osteopenia in 2010.^4^ By 2030, its prevalence is projected to increase to 71.4 million people.^4^ Furthermore, 80% of those affected by osteoporosis were postmenopausal women.^4^ Although data on the outcomes of dental and orthopaedic implants in osteoporotic patients are very limited, the compromised qualitative properties of the bone, strength, and healing associated with osteoporosis suggest these patients experience lower rates of implant success.

Healthy osseointegration is critically dependent on bone remodeling, which involves the reciprocal communication among osteoblasts, osteoclasts, mesenchymal stem cells (MSCs), and osteoclast precursors (OCPs).^5^, ^6^, ^7^ During remodeling, osteoclasts resorb a volume of bone leaving behind a foundation with a specific chemistry,^8^ stiffness,^9^ and morphology^10^ for osteoblasts to synthesize and calcify their matrix. Osteoclasts produce factors (both independent and as a byproduct of matrix resorption) capable of regulating MSC and osteoblast migration and their subsequent osteogenesis.^11^ In turn, MSCs and osteoblasts release factors capable of limiting the degree and extent to which osteoclasts resorb bone. Any defects in the coupling of bone resorption to bone formation has not only been implicated in impaired healing and osseointegration but also the onset and progression of osteoporosis. Although age‐related mechanisms contributing to osteoporosis may originate from accelerated bone resorption or impaired bone formation,^12^ these processes are not independent.

Bisphosphonates are commonly used to combat osteoporosis by targeting osteoclasts, slowing the rate and severity of bone resorption. These events translate to decreased bone turnover and increased BV and BMD.^13^ Bisphosphonates have also been reported to exert anabolic effects on osteoblasts *in vitro* by stimulating proliferation^14^, ^15^, preventing apoptosis,^16^, ^17^ and enhancing production of alkaline phosphatase, bone morphogenetic protein (BMP)‐2, type‐I collagen, and osteocalcin,^15^, ^18^, ^19^, ^20^ The positive effects bisphosphonates have on osteoblasts has provided some rationale for their use to enhance osseointegration. Other studies, however, have reported impaired mineralized bone nodule formation^21^, ^22^ and responses to parathyroid hormone (PTH) with bisphosphonates.^23^, ^24^, ^25^, ^26^, ^27^ Furthermore, bisphosphonates can impede angiogenesis^28^, ^29^, ^30^ and are associated with osteonecrosis of the jaw (ONJ) at high doses,^31^, ^32^ both of which can be detrimental to peri‐implant bone formation and osseointegration. Moreover, functional osteoclasts are integral to healthy bone remodeling. Therapeutic interventions targeting either half of this process will inevitably affect its counterpart, contraindicating the use of bisphosphonates when bone remodeling is of the utmost importance like implant osseointegration.

Considering the growing number of osteoporotic patients^33^ and high rate of bisphosphonate prescriptions,^34^ the success of implant outcomes and osseointegration in this demographic has turned into a significant dental and orthopaedic challenge. To optimize the use of implant technologies in patients with compromised bone structure and metabolism, a more complete understanding of the biological response to surface design and the impact of bisphosphonate treatments on osseointegration are needed. The goal of this study was to assess the effects post‐menopausal osteoporosis and bisphosphonate treatment have on the osseointegration of clinically used microstructured titanium (Ti) implants.

## 2. Materials and Methods

This study was conducted under approval of the Institutional Animal Care and Use Committee at Virginia Commonwealth University. All experiments were carried out in accordance with approved procedures and reported according to ARRIVE guidelines. All animals were treated humanely per the guidelines outlined in the Guide for the Care and Use of Laboratory Animals by the National Institutes of Health. Animals were single‐housed in an individually ventilated, solid‐bottomed polysulfone cage and kept at a temperature of 17–28 °C with a humidity of 40–70% and a 12/12 h light/dark cycle.

### 2.1 Implant Preparation

Ti implants were designed to fit a rat femur and provided by Institut Straumann AG (Basel, Switzerland). 3.5mm long implants with a 2.5mm outer diameter and a 0.8 mm pitch were initially machined from a rod of grade 4 Ti. They were then processed for 30 s in a 55°C 2% ammonium fluoride/2% hydrofluoric acid/10% nitric acid solution. Implants were sand‐blasted with large grit particulate (250–500 μm corundum) followed by acid etching in a boiling mixture of HCl and H_2_SO_4_ to generate implant with a surface similar to the clinically used SLA implant.^35^, ^36^ Implants were cleaned in HNO_3_, rinsed in ultrapure water, packed in aluminum foil, and γ‐irradiated before use.

### 2.2 Implant Characterization

#### 2.2.1. Scanning Electron Microscopy (SEM)

Scanning electron microscopy (SEM; Hitachi SU‐70 FE‐SEM, Hitachi, Tokyo, Japan) was used to qualitatively evaluate implant surface structure and roughness. Six images at varying magnifications were captured on 3 different SLA implants using 5 kV accelerating voltage for a total of 18 images.

#### 2.2.2. Laser Confocal Microscopy

Laser confocal microscopy (LCM, Zeiss LSM 710, Zeiss, Oberkochen, Germany) was used to quantitatively evaluate surface micro‐roughness. Measurements on each implant (*n* = 3) were taken over an area of 106.2 μm × 106.2μm with a 20× objective and a scanning pitch of 50 nm. A Gaussian high‐pass filter with a cutoff wavelength of 100 μm was used when calculating average surface roughness (S_a_) over three scans per implant (total *n* = 9).

#### 2.2.3. X‐Ray Photoelectron Spectroscopy (XPS)

Chemical composition of the samples (*n* = 3) was obtained from the sample surfaces by XPS (Thermo K‐Alpha XPS, Thermo Fisher Scientific, Waltham, MA, USA). Spectra were collected using a 500 μm spot size, using an XR5 gun and Al Kα x‐ray source at 15 kV. Scans were taken with a 20ms dwell time and 1eV step size. Three different locations on each sample (total *n* = 9) were analyzed.

### 2.3. Animals and Surgical Procedures

**Figure 1:**
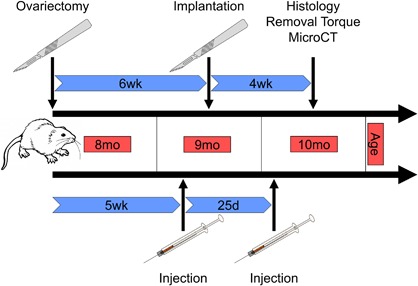
Schematic of the Experimental Procedures. 40, 8‐month old, skeletally mature, virgin, female CD Sprague‐Dawley rats underwent ovariectomy (OVX; *n* = 20) or sham OVX (SHOVX; *n* = 20) surgery. After 5 weeks, animals were injected with either ibandronate (BIS) or phosphate buffered saline (PBS) at a concentration of 25μg/kg/25days followed by insertion of a Ti SLA implant 1 week later. Implants were allowed to osseointegrate for 28d.

A schematic detailing the timing of surgical procedures and treatments is shown in figure 1. All surgical procedures were performed at the same session under isoflurane inhalation anesthesia. 40, 8‐month‐old, skeletally mature, virgin, female CD Sprague‐Dawley rats (Charles River Laboratories, Wilmington, MA) underwent ovariectomy (OVX; *n* = 20) or sham OVX (SHOVX; *n* = 20) surgery. The OVX and SHOVX surgeries were performed by Charles River Laboratories. Development of the osteoporotic phenotype occurred over the next 5 weeks.

#### 2.3.1. Bisphosphonate Treatment

Animals received two bisphosphonate injections. The first injection was at 5wk post‐OVX; animals received subcutaneous injections of either the bisphosphonate (BIS), ibandronate (AuroMedics Pharma LLC, Dayton, NJ, USA) (25 µg/kg), or phosphate buffered saline (PBS) resulting in four groups: 1) SHOVX + PBS (*n* = 10 animals); 2) SHOVX + BIS (*n* = 10 animals); 3) OVX + PBS (*n* = 10 animals); 4) OVX + BIS (*n* = 10 animals). One week after the first injection, the implants were placed (described below). Animals received a second injection of ibandronate or PBS 25 days after the first injection; thus animals had bisphosphonate treatment before and after the placement of the implants. Treatment method, dose, and frequency were based on previous reports detailing that total amount, not frequency, of ibandronate injections is important for drug efficacy with an optimal dose of 1.0µg/kg/day.^37^ A subcutaneous injection was chosen over oral administration to ensure total dosage delivery to each rat. 25 days was chosen to mimic monthly injections used clinically while also minimizing distress arising from frequent injections. This timing has also been successfully employed previously.^38^

#### 2.3.2. Transcortical Implant Surgeries

One week after the first injections, SLA modified implants were placed transcortically into the distal metaphysis of both femurs. Animals were anesthetized with 5% isoflurane gas inhalation. The hind limbs were prepared by shaving and cleaning using ethanol and chlorohexidine. Anesthesia was maintained at 4% isoflurane in O_2_ gas inhalation for the duration of the surgical procedure. Cleaned, anesthetized animals were placed in a supine position and covered with a sterile surgical drape. An 8mm incision was made over the medial side of the right knee. Overlying muscle was separated, and the distal femur was exposed using blunt dissection to a point immediately above the articular capsule. A high‐speed dental hand‐piece was used to create a progressively larger pilot hole in the distal femur using a series of increasing diameter drill bits (Ø1.0 mm, Ø1.6 mm, Ø2.0 mm, and Ø2.2 mm) with a 3.5mm drill stop. Ti SLA implants were then screwed into place by hand using a custom‐made driver. Following implant placement, custom‐made stainless steel cover screws were placed on the end of the implant to prevent bone from growing into the internal threading of the implant. The periosteum and muscle were reapposed and sutured in place using resorbable sutures and the skin was closed with 9mm wound clips. These steps were then repeated for the left femur. Animals recovered from anesthesia on a water‐circulating warming pad and were injected subcutaneously with 1mg/kg buprenorphine SR LAB. All animals had access to water *ad libitum* for the duration of the study; however, food access was regulated.

#### 2.3.3. Diet and Pair Feeding

The diet of ovariectomized animals is a potential source of at least two confounding variables. The first is the tendency of rats to have increased appetites following OVX. This can lead to excessive weight gain potentially altering the mechanical loading on the implant thus affecting the process of osseointegration.^39^ In order to eliminate weight gain as a confounding variable, the animals in this study were pair fed. Each week SHOVX+PBS animals had their food intake monitored by calculating the difference in available food weight in a 24 hour period. The average difference across the four SHOVX+PBS animals was given to each animal in the three remaining groups daily. The success of the pair feeding regimen was verified by weekly weighing of all animals for the duration of the study.

The content of the food can also confound the results studies involving ovariectomized animals. Many rodent feeds are made from products known to contain phytoestrogens. Phytoestrogens are structurally similar with 17β‐estradiol causing them to have estrogenic and/or anti‐estrogenic effects potentially preventing bone loss.^40^ Because the dietary estrogenic activity is a concern, all animals were given a phytoestrogen‐free diet (Advanced Protocol Verified Casein Diet 10 IF, LabDiet, St. Louis, MO, USA).

### 2.4. Tissue Analysis

Transcortical implants were allowed to osseointegrate for 28d, after which rats were euthanized via CO_2_ inhalation. The hind limbs from each animal were isolated for removal torque testing (*n* = 5 animals/group) or micro‐computed tomography (microCT) and histological analysis (*n* = 5 animals/group). Each limb was treated as a separate sample providing an effective sample size of 10 per group for both the removal torque testing and microCT/histological analysis.

#### 2.4.1 MicroCT

MicroCT (SkyScan 1173, Bruker, Kontich, Belgium) was used to assess the osteoporotic phenotype in the femoral head as well as evaluate peri‐implant bone growth and bone‐to‐implant contact (BIC) in the distal femur. Femurs used for microCT were stored and fixed in 10% neutral buffered formalin for at least 24hr prior to imaging. The femoral heads of fixed samples were scanned at a resolution of 1120 × 1120 pixels (image pixel size of 12.94 μm) over 360° using a 1.0 mm aluminum filter, 85kV voltage, 94 µA current, and 270 ms exposure time. 5 x‐ray projections were acquired every 0.2° and averaged. A standard Feldkamp reconstruction was done using NRecon Software (Bruker) with a beam hardening correction of 20% and a Gaussian smoothing kernel of 0. To calibrate for cortical tissue mineral density and trabecular bone mineral density, 4mm epoxy resin rods containing concentrations of 0.25 and 0.75 gcm^−3^ calcium hydroxyapatite (CaHA). Cortical and trabecular bone were isolated and densities determined separately using CTAn analysis software (Bruker). Total porosity and trabecular number were also quantified from the isolated trabecular bone.

The distal femoral metaphysis was scanned at a resolution of 1120 × 1120 pixels (image pixel size of 13.66 μm) over 360° using a 0.25mm brass filter, 120kV voltage, 66 µA current, and 420 ms exposure time. 5 x‐ray projections were acquired every 0.2° and averaged. After reconstruction, a uniform volume of interest (VOI) was isolated. The VOI began at the base of the implant and extended 3mm towards its apex to eliminate any variability arising from the implant not being exactly at bone level for every sample. The VOI was shrink‐wrapped, dilated 2 pixels around the implant, and subtracted from the original VOI. The remaining bone and implant were thresholded and quantified as the total bone volume (BV) and then normalized to the total uniform VOI (TV) to get bone volume over total volume (BV/TV). The BIC was calculated using a separate BV that encompassed only the bone in direct contact with the implant. The same VOI was dilated 10 pixels around the implant, rethresholded, further dilated by 3 pixels, and subtracted from the original VOI. After eroding the remaining VOI by 3 pixels, the remaining BV was normalized to the implant volume.

#### 2.4.2 Histology

Following imaging with microCT, samples were placed in fresh 10% neutral buffered formalin and sent to be commercially processed for calcified histological staining (Histion, Everett, WA, USA). Femurs were embedded in methyl methacrylate, sectioned longitudinally relative to the implant and transaxially relative to the femur (transcortical), and stained with Stevenel's blue and van Gieson. Sections were imaged using bright field light microscopy with an AxioCam MRc5 camera and Axio Observer Z1 and analyzed using ZEN 2012 Blue Edition software (Zeiss). Histomorphometry was then used to evaluate peri‐implant bone growth and BIC.

New peri‐implant bone growth was quantified within a uniform rectangular region of interest (ROI) for all samples that was 3.56 mm in width by 3.0 mm in length with an area of 10.7 mm^2^. The ROI was drawn 0.3 mm beneath the distal portion of the implant and centered. The area of all bone within the ROI was then quantified (BV) and normalized to the area of the ROI (TV). The area of bone contained within the ROI divided by the area of the ROI was defined as the histological BV/TV. In addition, the perimeter of the implant contained within both the trabecular region and the cortical region of the bone was measured. The trabecular BIC and cortical BIC were determined by dividing the length of bone in direct contact with the implant by the trabecular and cortical perimeter length respectively. The total BIC was calculated by summing both lengths of contact and dividing by the total perimeter of the implant.

#### 2.4.3 Removal Torque

Removal torque testing was performed on fresh, non‐fixed samples using an ElectroForce 3200 Series III test instrument (TA Instruments, New Castle, DE, USA). Because of their asymmetrical shape, femurs were mounted in 1cm diameter flexible polyurethane tubing to ensure no movement of the femur during analysis (Fig. 6A). The tubing was cut into 5cm segments and halved longitudinally to provide access to the transcortical implant. Femurs were secured to the tubing with polyurethane adhesive and allowed to cure overnight at 4°C prior to testing. The transcortical implant in each hind limb was then fit to a custom‐made driver and aligned to the testing machine axis to ensure no initial torque was present on the implant (0Nm). A clamp was then carefully tightened, securing each sample in place with no initial compressive load present on the implant (0Nm). A 0Nm compressive load was critical to guarantee no axial mismatch between the implant and the testing apparatus, which could greatly alter our results. Torque was then applied to each sample with a rotational speed of 0.1°s^−1^ with an axial displacement of 0.8 mm/360° to ensure no compressive load was applied to the sample during the duration of each test. Torque vs. degree graphs were generated for each sample and fit to a bilinear model in order to distinguish the toe‐region from the linear region using an open‐source least squares spline modeling package (SLM − Shape Language Modeling version 1.14) for MATLAB (MathWorks, Natick, MA, USA). The linear region of each graph was then evaluated for the maximum torque, torsional energy (area below linear region), and torsional stiffness (slope of linear region).

### 2.5 Osteoblast Response In Vitro

To further assess the effects of bisphosphonate treatment, following euthanasia, calvarial osteoblasts were isolated from the frontal and parietal bones of rats in each of the four experimental groups. After removal of the periosteum and soft tissue, bone fragments were digested for 15 min at 37°C with 0.25% trypsin‐EDTA (Life Technologies, Carlsbad, CA). Bones were minced into pieces approximately 1mm x 1mm and placed into a 100 mm × 20 mm Petri dish with Dulbecco's modified Eagle medium (DMEM; Mediatech, Manassas, VA) + 10% fetal bovine serum (FBS) + 1% penicillin‐streptomycin (Life Technologies). At confluence, cells were subpassaged and cultured as above. The osteoblast phenotype of each of the four groups of isolated cells was confirmed by measuring alkaline phosphatase specific activity and osteocalcin production after treatment of confluent cultures with either 0M or 10^−8^M 1α,25‐dihydroxy vitamin D_3_ (1α,25(OH)_2_D_3_; Enzo Biochem, Farmingdale, NY) for 24 hrs (Supplementary Figure  1) on tissue culture polystyrene (TCPS).

Validated rat osteoblasts (rOBs) from each experimental group were cultured on TCPS or 15 mm Ti SLA disks. Disks were prepared from Ti sheets, but subjected to the same sand‐blasting and acid etching procedure as described above. Cells were plated at a density of 10,000 cells/cm^2^ and incubated at 37°C in an atmosphere of 5% CO_2_ and 100% humidity. Media were changed 24 h after plating and every 48h thereafter for 7d. At 7d, cells were incubated with fresh DMEM for 24 h. Media were collected and immunoassays were used to measure levels of intact rat osteocalcin (Alfa Aesar, Haverhill, MA) rat/mouse osteopontin (R&D Systems, Minneapolis, MN), mouse osteoprotegerin (R&D Systems), human/mouse/rat BMP2 (PeproTech), mouse receptor activator of nuclear kappa‐B ligand (RANKL; R&D Systems), and rat vascular endothelial growth factor‐A (VEGF; R&D Systems).

After collection of media, cell monolayers were washed twice with 0.2 ml PBS, lysed in 0.05% Triton X‐100, and homogenized by sonication at 40 amplitude using a Vibra‐Cell ultrasonicator (Sonics & Materials Inc., Newtown, CT). DNA content in the cell layer lysate was measured with PicoGreen (Promega, Madison, WI) using a Synergy H1 Hybrid Reader fluorescence detector (BioTek, Winooski, VT) at an excitation of 485 nm and emission of 538 nm. Alkaline phosphatase specific activity [orthophosphoric monoester phosphohydrolase, alkaline; E.C. 3.1.3.1] was assayed by measuring the conversion of *p*‐nitrophenylphosphate to *p*‐nitrophenol at pH 10.25 and temperature of 37°C. Absorbance was measured at 405nm. Activity was normalized to total protein content in the cell lysates as determined by bicinchoninic acid protein assay kit (Thermo Fisher Scientific, Waltham, MA). Immunoassay data were normalized to DNA content.

### 2.6. Statistical Analysis

Based on previous studies,^39^, ^41^ in order to detect a 30% mean difference with 20% variance and a type I error rate of 0.05, a two‐tailed one‐way ANOVA power analysis determined a sample size of 10 per group is necessary to maintain 80% power. In order to ensure that differences in mechanical loading would not affect the results, identical implants were placed in the right and left hind limbs. In addition, animals received the same systemic treatment (e.g. OVX/SHOVX or ibandronate/PBS), which prevents any potential carry‐over effects between legs. Since both legs were treated identically, it is safe to assume that movement or loading in one limb will not affect the movement or loading experienced by the other limb. The design of our study permits the assumption that implants from individual limbs can be treated as independent data points rather than dependent. Data are presented as the mean ± standard error (SE) for each analysis. All cell culture experiments had a sample size of six (*n* = 6) and repeated at least three times to ensure validity of the results. Data shown in the figures are from representative experiments. A one–way analysis of variance with a two‐tailed Tukey correction was performed to adjust for multiple comparisons to maintain an experiment‐wise error rate (α) of 0.05. All statistical analyses were performed using JMP statistical software (SAS Institute, Cary, NC, USA).

## 3. Results

### 3.1. Transcortical Implant Characterization

**Figure 2:**
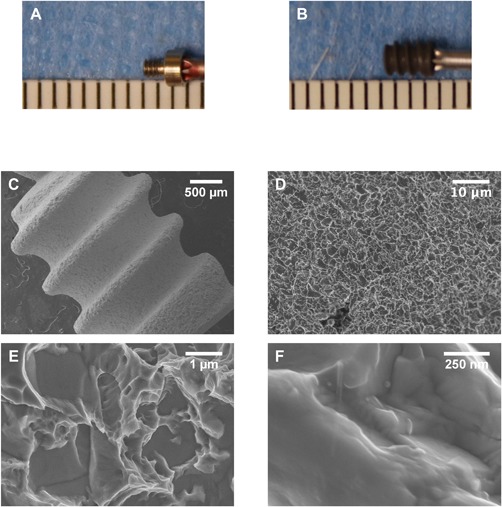
Qualitative Characterization of Ti SLA Implants. Photographs showing the dimensions of the stainless steel cover screw (A) and Ti SLA implant (B). The space between each mark represents 1mm. Surface morphology was assessed using scanning electron microscopy (C − D).

Qualitative images of the transcortical Ti SLA implants as well as the cover screw are shown in Figure 2. Gross morphology of the stainless steel cover screw and Ti SLA implant can be seen in Figure 2A and Figure 2B respectively. SEM images of the transcortical Ti SLA implants (Fig. 2C–F) reveal the rough surface induced by the sand‐blasting and acid etching procedure, resulting in a combination of microscale and submicron‐scale surface features. These complex features were also strewn with unmodified areas characterized by flat and smooth sections. Together the sand‐blasting and acid etching procedure led to an average surface roughness (S_a_) of 3.91 ± 0.09 μm as measured by confocal microscopy (Table 1). XPS survey spectra (Table 1) displayed titanium (Ti), oxygen (O), and carbon (C) as the main atomic components of the SLA implant.

**Table 1:** Average roughness (S_a_) and atomic concentrations (%) of SLA implant surfaces as measured by confocal microscopy and XPS

	Confocal Microscopy	X‐Ray Photoelectron Spectroscopy		
	Mean Roughness (S_a_) ± [μm]	O (%)	C (%)	Ti (%)
SLA	3.91 ± 0.09	38.19 ± 1.37	51.83 ± 1.01	9.89 ± 0.21

### 3.2. Evaluation of Pair Feeding and Osteoporotic Phenotype

Animals from the four groups maintained similar weights and levels of activity throughout the duration of the study. Select week results from the overall ANOVA F‐test (Week 1: F_3,36_ = 1.5, *p* = 0.2376; Week 4: F_3,36_ = 1.1, *p* = 0.3467; Week 8: F_3,36_ = 0.6, *p* = 0.6041; Week 10: F_3,36_ = 0.2, *p* = 0.8946) indicate a successful pair feeding regimen.

**Figure 3:**
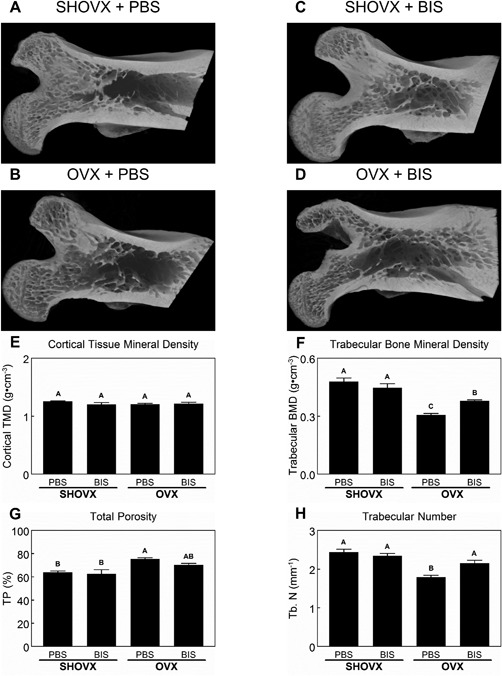
Characterization of the Ovariectomy Induced Osteoporotic Phenotype. 8 month old, female, virgin, CD Sprague Dawley rats underwent sham ovariectomy (SHOVX) or ovariectomy (OVX) surgery. After 5 weeks, animals were injected with either ibandronate (BIS) or phosphate buffered saline (PBS) at a concentration of 25 μg/kg/25days followed by insertion of a Ti SLA implant 1 week later. After 28d of osseointegration, femurs were isolated and placed in 10% formalin. Femoral heads of the animals were analyzed with 3D microCT reconstructions (A–D). Cortical tissue mineral density (E), trabecular bone mineral density (F), total porosity (G), and trabecular number (H) were quantified from the microCT reconstructions. Data shown are the mean ± standard error (SE) of ten (*n* = 10) independent samples. Groups not sharing a letter are statistically significant at an α = 0.05.

MicroCT was used to evaluate the osteoporotic phenotype of the femoral head with representative images shown in Figure 3A–D. The cortical tissue mineral density (Fig. 3E) was not affected by the OVX nor treatment with ibandronate. However, the trabecular bone mineral density (Fig. 3F) was significantly reduced in OVX animals compared to SHOVX animals. Treatment with ibandronate mitigated the loss of the trabecular bone mineral density. Total porosity (Fig. 3G) was increased in OVX animals receiving PBS compared to SHOVX animals. OVX animals receiving ibandronate had a total porosity similar to both SHOVX animals and OVX animals receiving ibandronate. Trabecular number (Fig. 3H) decreased in OVX animals receiving PBS compared to the other groups.

### 3.3. MicroCT

**Figure 4:**
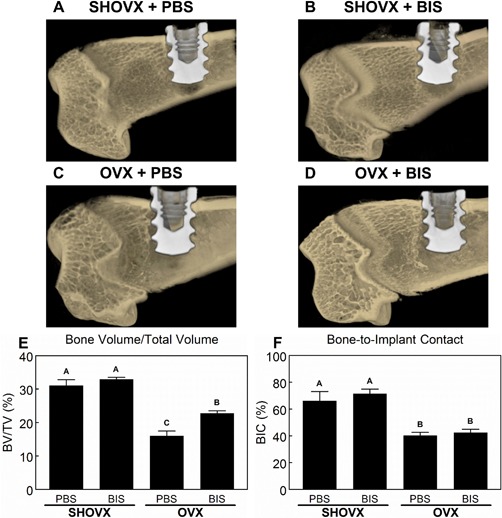
MicroCT Assessment of Implant Osseointegration. 8 month old, female, virgin, CD Sprague Dawley rats underwent sham ovariectomy (SHOVX) or ovariectomy (OVX) surgery. After 5 weeks, animals were injected with either ibandronate (BIS) or phosphate buffered saline (PBS) at a concentration of 25μg/kg/25days followed by insertion of a Ti SLA implant 1 week later. After 28d of osseointegration, femurs were isolated and placed in 10% formalin. Distal femurs of the animals were analyzed with 3D microCT reconstructions (A–D). Bone volume over total volume (BV/TV; E) and bone‐to‐implant contact (BIC; F) were quantified from the microCT reconstructions. Data shown are the mean ± standard error (SE) of ten (*n* = 10) independent samples. Groups not sharing a letter are statistically significant at an α = 0.05.

3D reconstructions of microCT scans (Fig.4A–D) showed peri‐implant bone formation in all animal groups. Furthermore, the transcortical implants did not contact the growth plate eliminating any potential influence on the bone formation in our defined VOI. The BV/TV (Fig. 4E) decreased in OVX animals compared to SHOVX, and the severity of the reduction was lessened in OVX animals receiving ibandronate. Osseointegration was achieved in all animal groups as well. BIC values (Fig. 4F) obtained through microCT analysis were reduced in OVX animals compared to SHOVX animals after 4 weeks. Ibandronate treatment of both SHOVX and OVX animals did not influence BIC values.

### 3.4. Histology

**Figure 5:**
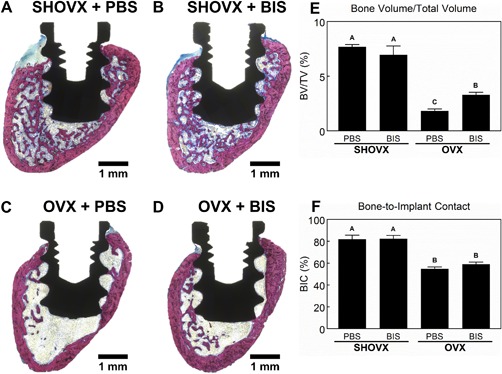
Histological Assessment of Implant Osseointegration. 8 month old, female, virgin, CD Sprague Dawley rats underwent sham ovariectomy (SHOVX) or ovariectomy (OVX) surgery. After 5 weeks, animals were injected with either ibandronate (BIS) or phosphate buffered saline (PBS) at a concentration of 25 μg/kg/25days followed by insertion of a Ti SLA implant 1 week later. After 28d of osseointegration, femurs were isolated and placed in 10% formalin. Distal femurs of the animals were embedded in methyl methacrylate, sectioned longitudinally relative to the implant and transaxially relative to the femur (transcortical), and stained with Stevenel's blue and van Gieson (A − D). Bone volume over total volume (BV/TV; E) and bone‐to‐implant contact (BIC; F) were quantified using histomorphometrics. Data shown are the mean ± standard error (SE) of ten (*n* = 10) independent samples. Groups not sharing a letter are statistically significant at an α=0.05.

Histological sections of transcortical implants from each animal group (Fig. 5A–D) confirmed the results seen from the microCT analysis. More trabecular bone can be seen in the SHOVX groups (Fig. 5A, B) compared to the OVX groups (Fig. 5C, D). Quantification of the total bone area with a fixed region of interest (Fig. 5E) was significantly higher in SHOVX animals compared to OVX animals. Administration of ibandronate mitigated some of the loss in OVX animals. BIC (Fig. 5F) was higher in SHOVX compared to OVX animals and were not influenced by ibandronate treatment.

### 3.5. Mechanical Testing

**Figure 6:**
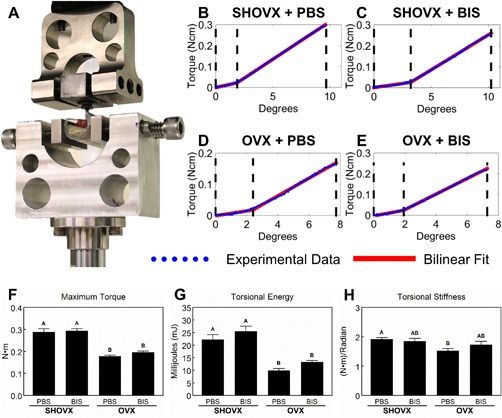
Removal Torque Assessment of Implant Osseointegration. 8 month old, female, virgin, CD Sprague Dawley rats underwent sham ovariectomy (SHOVX) or ovariectomy (OVX) surgery. After 5 weeks, animals were injected with either ibandronate (BIS) or phosphate buffered saline (PBS) at a concentration of 25μg/kg/25days followed by insertion of a Ti SLA implant 1 week later. After 28d of osseointegration, femurs were isolated and analyses were performed on fresh, non‐fixed samples as outlined (A). Torque vs. degree graphs were generated for each sample and fit to a bilinear model in order to distinguish the toe‐region from the linear region (B–E). The linear region of each graph was then evaluated for the maximum torque (F), torsional energy (G), and torsional stiffness (H). Data shown are the mean ± standard error (SE) of ten (*n* = 10) independent samples. Groups not sharing a letter are statistically significant at an α=0.05.

Isolated femurs were secured in polyurethane tubing and aligned to the machine axis in a custom‐fabricated sample holder to ensure no movement was created during the test (Fig. 6A). Representative torque vs. degree graphs for each group (Fig. 6B–E) display the bilinear model (red) fit to the experimental data (blue). The end of each curve identifies failure, and no secondary peaks were observed for any sample. The middle vertical dashed line (black) separates the toe region (left section) and the linear region (right section) as determined by the least squares spline modeling package in MATLAB. The maximum torque (Fig. 6F) and torsional energy (Fig. 6G) was greatest in SHOVX animals and significantly reduced in OVX animals. Ibandronate treatment had no effect on either parameter. Torsional stiffness (Fig. 6H) was greatest in SHOVX animals treated with PBS and lowest in OVX animals treated with PBS. SHOVX+BIS and OVX+BIS animal torsional stiffness values were not different from either SHOVX+PBS or OVX+PBS.

### 3.6 In Vitro Response of Calvarial Osteoblasts

**Figure 7:**
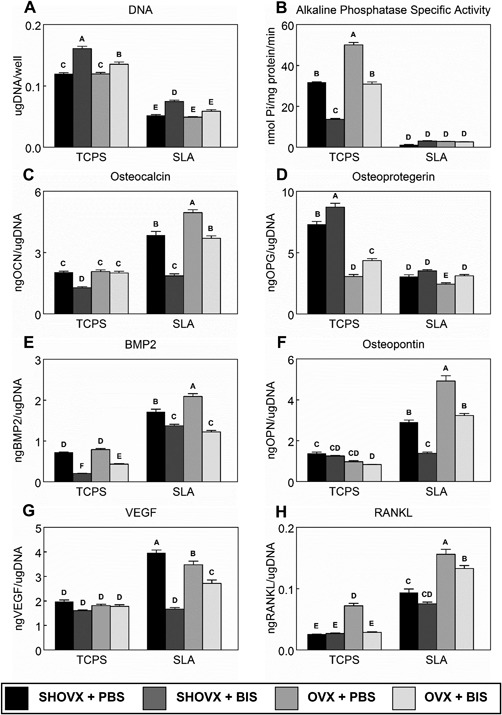
In Vitro Response of Primary Calvarial Osteoblasts. Calvarial osteoblasts were isolated from each of the four groups of animals and cultured separately on either TCPS or SLA in DMEM. After 7d, cells were treated with fresh DMEM for 24 h. After 24 h, media were collected and cell lysates were assayed for DNA content (A) and alkaline phosphatase specific activity (B). Media were assayed for osteocalcin (C), osteoprotegerin (D), BMP2 (E), osteopontin (F), VEGF (G), and RANKL (H). Data shown are the mean ± standard error (SE) of six (*n* = 6) independent samples. Groups not sharing a letter are statistically significant at α=0.05.

1α,25(OH)_2_D_3_ stimulated osteocalcin production and alkaline phosphatase specific activity in confluent cultures of rOBs from all four experimental groups, confirming their successful isolation and expansion (Supplementary Fig.  1). DNA content (Fig. 7A) and alkaline phosphatase specific activity (Fig. 7B) in cultures grown on SLA were lower than in cultures grown on TCPS. DNA content was higher in cultures of osteoblasts isolated from ibandronate treated animals grown on TCPS compared to the other treatment groups. Among SLA cultures, DNA content was highest in SHAM+BIS osteoblasts. Ibandronate treatment decreased alkaline phosphatase specific activity among TCPS cultures, but no effect was observed in SLA cultures. SLA increased osteoblast osteocalcin (Fig. 7C) production in all groups except SHAM+BIS, which remained similar to TCPS cultures. Furthermore, OVX+BIS osteoblasts had decreased osteocalcin production compared to OVX+PBS osteoblasts. Osteoprotegerin (Fig. 7D) levels decreased in SLA cultures compared to TCPS. Osteoblasts isolated from OVX animals had decreased osteoprotegerin levels in TCPS cultures, although ibandronate treatment did stimulate its production. Production of BMP2 (Fig. 7E), osteopontin (Fig. 7F), VEGF (Fig. 7G), and RANKL (Fig. 7H) was increased on SLA compared to TCPS with the highest levels of BMP2, osteopontin, and RANKL occurring in OVX+PBS osteoblasts. The highest levels of VEGF were observed in SHAM+PBS osteoblasts. Ibandronate treatment led to decreased productions of these proteins in SLA cultures compared to their respective PBS controls with SHAM+BIS osteoblasts producing amounts of osteopontin and VEGF to levels similar to those observed in TCPS cultures.

## 4. Discussion

Osseointegration involves a complex cascade of biological events, ultimately leading to the structural and functional connection between mature, lamellar bone and the inserted implant.^3^ The cellular miscommunication associated with osteoporosis leads to excessive bone resorption and impaired bone remodeling, which challenges successful osseointegration and implant outcomes. Although bisphosphonates are known to increase BV and BMD by mitigating the resorptive damage caused by overactive osteoclasts, their continued disruption of bone remodeling may contribute to greater risks of compromised implant osseointegration. In order to improve osseointegration and long‐term stability of implants in osteoporotic patients, we sought to determine the effects ibandronate treatment has on the osseointegration of microrough Ti implants in an aged, OVX‐induced osteoporotic rat model.

In the present study, microCT analyses, calcified histomorphometrics, and removal torque testing revealed decreased osseointegration in ovariectomized animals compared to SHAM operated controls. Moreover, ibandronate treatment did not affect BIC and removal torque values, although it was able to halt the progression of the osteoporotic phenotype as observed through increased BV/TV in ovariectomized animals. The physiological consequences of osteoporosis are known to alter cellular communication leading to lower qualitative properties of the bone and impaired healing.^42^ It is clear from this study and others that the estrogen deficient osteoporotic phenotype negatively impacts implant osseointegration.^43^, ^44^, ^45^, ^46^ Previous studies, however, have lauded the use of bisphosphonates to reverse the negative effects of osteoporosis on implant osseointegration.^47^, ^48^, ^49^, ^50^, ^51^, ^52^, ^53^ On the other hand, BIC and removal torque values were either unaffected^52^, ^54^ or decreased^55^ by bisphosphonate therapy in healthy animals. Despite the inhibitory effect bisphosphonates have on bone remodeling, the precise mechanism of action is not fully understood, which has led to many studies with variations in experimental methods. Selection of the appropriate animal model, development of the osteoporotic phenotype, timing of both the bisphosphonate treatment and implant insertion as well as the route of bisphosphonate administration and location of implant placement are important factors. Conflicting results between these studies and ours can be largely explained through these differences. It should also be noted that the femoral implant model is not an exact replica of bone turnover in the oral cavity, which could limit the translation of our results to clinical outcomes for patients receiving dental implants rather than orthopaedic implants.

An ovariectomized rat is a well‐characterized model that closely mimics postmenopausal osteoporosis in humans. Basic multicellular unit‐based endocortical and cancellous bone remodeling occurs in both rats and humans.^56^, ^57^ Estrogen deficiency in both humans and rats leads to an increased presence of osteoclasts on the endocortical bone surface causing cancellous and endocortical bone loss by altering the balance between bone formation and bone resorption.^58^, ^59^, ^60^ In contrast, there is increased bone formation at the periosteal surface.^58^, ^61^ As a result of the opposing changes in radial growth and endocortical remodeling, cortical bone volume generally remains unchanged in ovariectomized rats, similar to what was observed in this study. However, trabecular bone turnover is known to be elevated in ovariectomized rats,^62^, ^63^ which explains the increased total porosity as well as the decreased trabecular number in PBS treated ovariectomized rats.

Evidence also suggests that both the age of the animal during OVX and implant insertion could significantly influence the results. While rats reach skeletal maturity around 3 months, their growth rate changes continuously for the first 6 months of life.^64^ In some instances, bone elongation in rats may persist up to their first 8 months.^64^ OVX of growing rats results in cancellous osteopenia due to increased resorption of growth plate calcified cartilage.^59^ The amount of primary spongiosa that serves as a template for future bone apposition is subsequently decreased.^59^ As bone growth slows with age, the contribution of altered endochondral ossification to the skeletal effects of OVX diminishes, and the contribution of altered bone remodeling increases and eventually becomes the predominant mechanism for the alteration of cancellous bone mass.

Further methodological complications arise when bone remodeling targeting therapies and implant osseointegration are introduced to the ovariectomized rat model. Many previous reports using this model to investigate implant osseointegration and bisphosphonate therapy perform the OVX surgery in rats under the age of 6 months.^47^, ^48^, ^49^, ^50^, ^51^, ^52^, ^53^ In spite of not approximating the relative timing of the onset of menopause in humans, it is difficult to attribute the impaired osseointegration to altered bone remodeling. A few of these studies do refer to the ovariectomized skeletal phenotype as estrogen‐deficient osteoporosis rather than postmenopausal; however, no attempt was made to control for the amount of phytoestrogens present in the animal diet, potentially altering the results. Another study using ovariectomized 6–9 month old retired breeders found that osseointegration of implants was enhanced by bisphosphonate therapy.^65^ The multiple pregnancies and lactations incurred by the retired breeders has been shown to result in osteopenia.^66^ Compared to age‐matched virgins, OVX of retired breeders results in less bone loss and more variable indices of bone mass and turnover.^66^

Regardless of the age at OVX, the osteoporotic phenotype needs sufficient time to develop as the combination of rat age and skeletal site used influence the resulting temporal pattern of bone loss.^67^ Studies treating animals with bisphosphonates^50^ or inserting implants^49^ on the same day as the OVX surgery do not fully capture the impact the physiological changes the osteoporotic phenotype has on implant osseointegration. Furthermore, studies treating animals with bisphosphonates either on the same day of the implant surgery or immediately after are not appropriate models to study the effects postmenopausal osteoporosis have on the osseointegration of implants. Clinically, patients diagnosed with osteoporosis will not remain untreated until after a surgical intervention.

The effects of bisphosphonates have also been shown to be dose‐dependent and vary with the chemistry of the compound being tested. Daily and weekly treatment regimens are available for many of these bisphosphonates, but it is predicted that novel and simplified regimens with bisphosphonates given at intervals between doses of longer than 1 week will be more convenient for patients and improve adherence to therapy. Ibandronate has the potential to be effective when administered less frequently than once weekly. Preclinical studies using OVX rats found that the lowest dose of ibandronate that completely prevented bone loss was 1 μg/kg/day. Although the bone turnover in skeletally mature rats is approximately 3–5 times higher compared to humans, it decreases with age and OVX.^60^, ^68^ Considering the age at OVX (8 months), the bone turnover of the rats used in this study closely reflects that of adult human bone. In addition, intermittent and continuous dosing of ibandronate produced similar results, so administration of 25 μg/kg/25 days was selected to mimic monthly injections patients would receive clinically as well as minimizing animal distress.^69^

3.5 mm long and 2.5 mm wide, grade 4, Ti implants were custom made for this study. This design allowed for a transcortical insertion, which more closely mimics clinical procedures as opposed to intramedullary canal insertion commonly performed on mice. The SLA surface modification is clinically used, and no studies have investigated this topography in response to either OVX induced osteoporosis and/or ibandronate treatment. Furthermore, this is the first reported use of these transcortical implants custom‐made to fit rat femurs. Scanning electron microscopy, laser confocal microscopy, and x‐ray photoelectron spectroscopy revealed a surface morphology, average surface roughness, and surface composition similar to what has been previously reported for implants processed in the same manner.^70^, ^71^, ^72^

Primary calvarial osteoblasts were obtained from the osteoporotic animals and were used to evaluate potential phenotypic and/or proliferative changes. rOBs were cultured on 15mm diameter and 1mm thick Ti disks that were processed to have the same surface microstructural features as those seen on the implants used *in vivo*. Both OVX and ibandronate treatment were able to condition rOBs *in vivo* to alter their response *in vitro*. Typically, cells cultured on rough surfaces display attributes of more differentiated and mature osteoblasts with decreased proliferative capacity and cell number than those on TCPS.^73^ While this was true for all groups, rOBs isolated from untreated OVX animals had higher productions of osteogenic markers compared to SHOVX, while bisphosphonate treatment decreased osteogenic and angiogenic markers compared to PBS treated cells. This suggests that the altered bone remodeling in OVX animals may be reinvigorated with implant surface properties, and bisphosphonate exposure may jeopardized the pro‐osteogenic response osteoblasts have to microstructured surfaces. Furthermore, components of the osteoprotegerin/RANKL/RANK system, a critical pathway for the regulation of bone remodeling, were affected differently. In this study, rOBs displayed a decreased production of osteoprotegerin and an increased production of RANKL compared to TCPS. Previous studies have shown that aging in humans^74^, ^75^ and rodents^76^, ^77^ leads to increased RANKL and decreased osteoprotegerin production by osteoblasts, which could be exacerbated by the enhanced maturation that Ti surfaces facilitate in osteoblasts. Interestingly, ibandronate treatment facilitated an increased osteoprotegerin and decreased RANKL productions indicating bisphosphonate treatment is able to influence and condition a shift from bone resorption to bone formation at the cellular level. Bisphosphonates have been shown to increase serum osteoprotegerin levels and that increases of osteoprotegerin correlate with increases in BMD.^78^ It is unclear if the increased osteoprotegerin reflects a direct effect of bisphosphonates on osteoblasts or an indirect effect by altering osteoclastogenesis and thereby altering the catabolism of osteoprotegerin.

In conclusion, our results show that osseointegration is decreased in osteoporotic animals. Clinically relevant doses of ibandronate were able to halt the progression of the osteoporotic phenotype. However, these doses were unable to enhance the osseointegration of microrough titanium implants. These *in vivo* results were supported by *in vitro* studies examining the biological response of primary rat osteoblasts to SLA disks.

## Supporting information

Supplemental Data have been included with the submission.

Supporting Figure S1.
