# Supplementary material for: Ibandronate Treatment Before and After Implant Insertion Impairs Osseointegration in Aged Rats with Ovariectomy Induced Osteoporosis
Source: JBMR Plus. 2019 Mar 6;3(7):e10184. doi: 10.1002/jbm4.10184 (PMC6659452; doi:10.1002/jbm4.10184)
Supplement: Supplementary file 1 — Supporting Figure S1. [file JBM4-3-na-s001.docx]

**
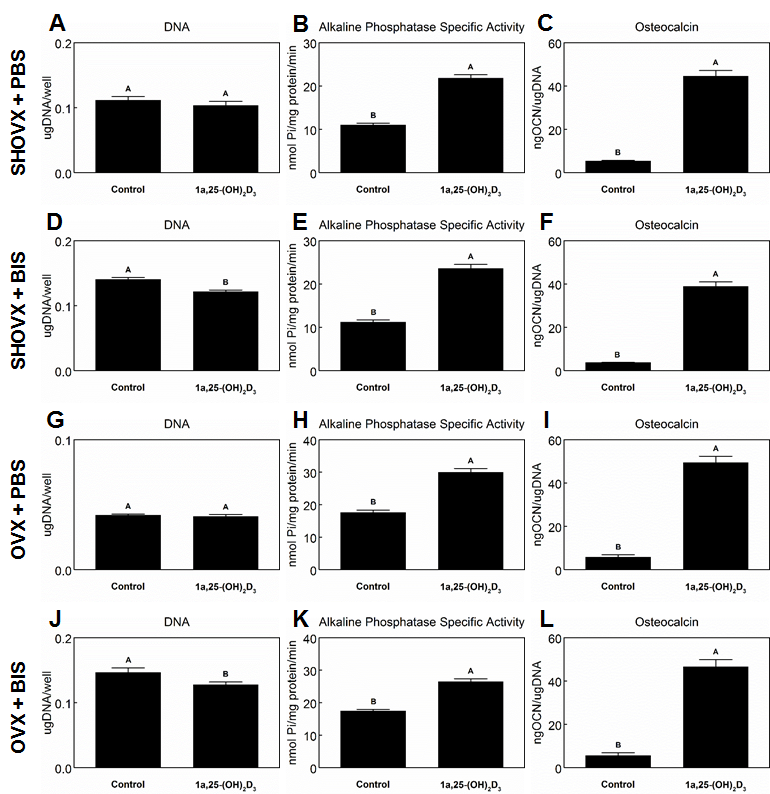
Supplementary Figure 1. Phenotype Confirmation of Isolated Calvarial Osteoblasts.** Calvarial osteoblasts isolated from each of the four groups of animals and cultured separately on TCPS. At confluence, cells were treated with fresh DMEM supplemented with vehicle (control) or 10^-8^M 1α,25(OH)_2_D_3_ for 24hrs. After 24h, media were collected and cell lysates of SHOVX + PBS osteoblasts (A – C), SHOVX + BIS osteoblasts (D – F), OVX + PBS osteoblasts (G – I), and OVX + BIS osteoblasts (J – L) were assayed for DNA content and alkaline phosphatase specific activity. Media were assayed for osteocalcin. Data shown are the mean ± standard error (SE) of six independent samples. Groups not sharing a letter are statistically significant at α=0.05.
